# Digital decision support for structural improvement of melanoma tumor boards: using standard cases to optimize workflow

**DOI:** 10.1007/s00432-024-05627-3

**Published:** 2024-03-08

**Authors:** David Hoier, Carolin Groß-Ophoff-Müller, Cindy Franklin, Michael Hallek, Esther von Stebut, Thomas Elter, Cornelia Mauch, Nicole Kreuzberg, Philipp Koll

**Affiliations:** 1 Department I of Internal Medicine, Center for Integrated Oncology Aachen Bonn Cologne Duesseldorf, University of Cologne, Faculty of Medicine and University Hospital Cologne, Cologne, Germany; 2 Department of Dermatology and Venereology, Center for Integrated Oncology Aachen Bonn Cologne Duesseldorf, University of Cologne, Faculty of Medicine and University Hospital Cologne, Cologne, Germany; 3 Kliniken der Stadt Köln gGmbH, Lung Clinic, Cologne, Germany; 4 Department of Dermatology, Venereology and Allergology, Ruhr-University Bochum, Bochum, Germany

**Keywords:** Malignant melanoma, Algorithm, Multidisciplinary tumor board (MDT), Tumor board evaluation, Digital recommendations, Documentation burden, Digital health, Mobile application, Expert-curated decision support system, Oncology

## Abstract

**Purpose:**

Choosing optimal cancer treatment is challenging, and certified cancer centers must present all patients in multidisciplinary tumor boards (MDT). Our aim was to develop a decision support system (DSS) to provide treatment recommendations for apparently simple cases already at conference registration and to classify these as “standard cases”. According to certification requirements, discussion of standard cases is optional and would thus allow more time for complex cases.

**Methods:**

We created a smartphone query that simulated a tumor conference registration and requested all information needed to provide a recommendation. In total, 111 out of 705 malignant melanoma cases discussed at a skin cancer center from 2017 to 2020 were identified as potential standard cases, for which a digital twin recommendation was then generated by DSS.

**Results:**

The system provided reliable advice in all 111 cases and showed 97% concordance of MDT and DSS for therapeutic recommendations, regardless of tumor stage. Discrepancies included two cases (2%) where DSS advised discussions at MDT and one case (1%) with deviating recommendation due to advanced patient age.

**Conclusions:**

Our work aimed not to replace clinical expertise but to alleviate MDT workload and enhance focus on complex cases. Overall, our DSS proved to be a suitable tool for identifying standard cases as such, providing correct treatment recommendations, and thus reducing the time burden of tumor conferences in favor for the comprehensive discussion of complex cases. The aim is to implement the DSS in routine tumor board software for further qualitative assessment of its impact on oncological care.

## Introduction

Everyday dermato-oncologists face the challenge to ensure evidence-based best clinical practice treatment for their cancer patients. Continuously updated treatment recommendations lead to an almost incomprehensible number of treatment options (Iqvia 2022). In malignant melanoma, we are faced with a rapidly changing therapeutic landscape in which immunotherapeutics and targeted treatment options are the gold standard in palliative and curative treatment (DKG 2020). Other potentially new emerging fields are neoadjuvant treatment approaches and adapted surgical procedures at earlier cancer stages (Amaria 2022; Luke 2022).

However, there are often deviations from the treatment considered best clinical practice and it must be assumed that a significant proportion of cancer patients do not receive optimal care (Bierbaum 2020; Bierbaum 2023; Heins 2017). An evaluation of the National Cancer Database (USA) on melanoma treatment showed, for example, that approx. 20% of patients with T2/3 melanoma were not treated according to the guidelines (Narang 2021).

In order to improve the quality of oncological care in Germany, the certification of cancer centers was implemented and has since become increasingly mandatory. Current data shows that treatment in certified cancer centers leads to higher treatment quality and better survival rates (Beckmann 2011; Dkg 2019; Kowalski 2022; Modabber 2021; Schmitt 2022; Wolff 2017).

A central feature for certification as a cancer center by the German Cancer Society (DKG) is the presentation of all treated melanoma patients from Stage IIC in a multidisciplinary tumor board (MDT) (Kowalski 2017). At the skin cancer center Cologne all melanoma cases from stage IIB have to be presented to MDT.

However, the MDT presentation of all tumor patients due to certification requirements led to a significant quantitative increase in case discussions. Studies have shown that this does not automatically translate to an increase in quality (Soukup 2016; Walraven 2019).

A large number of cases to be discussed pose a challenge to the participants in these boards (Soukup 2016; Walraven 2019). Data evaluating the extent to which MDT improves outcomes remain mixed and some studies have found a survival benefit, while others have found no such advantage. A 2019 analysis indicated that the 5 year survival rate was 15.6% higher among cases in well-organized MDT but almost 20% lower in disorganized MDT compared with no MDT (Keating 2013; Kesson 2012; Lu 2019; Stone 2020; Wong 2022).

In the end, the quality of MDT’s depends on the expertise and motivation of the participants and the time available to discuss the individual cases (Jalil 2018).

Decision support systems (DSS) could provide useful support here and appear to offer remarkable potential (Chen 2016; Letzen 2019; Soukup 2019). Surprisingly, however, artificial intelligence (AI)-based DSS have so far failed to gain acceptance in the field of clinical oncology (Bungartz 2018). This is even the case for standard oncological questions regarding first-line therapy (Schmidt 2017).

Expert-curated DSS algorithms, developed on knowledge base provided by oncological professionals, might be a superior approach for adequate decision support. As described in Nature Biotechnology in 2018, these expert-curated DSS provide multiple advantages when compared with AI-based systems (Bungartz 2018). Most importantly, expert-curated systems seem to represent clinical reality better than the AI-based systems used so far.

The aim of the present study was to evaluate the potential benefit of a DSS to optimize the workflow of tumor conferences.

## Materials and methods

We first created a query that simulated a melanoma tumor conference registration and requested all the information needed to provide a treatment recommendation. This algorithm was then implemented in the established oncology smartphone application "EasyOncology" (EO). This certified medical product is aimed at specialized personnel and provides the treatment concepts for the most common tumor entities. Thus, the basis was provided to easily match the smartphone DSS recommendations with the real decisions of the MDT and to determine the quality of the concordance.

To evaluate the reliability of each DSS recommendation, we followed the same approach that was used to assess the accuracy of AI-based DSS and benchmarked the digital treatment recommendations against real-world MDT decisions (Choi 2019; Kim 2019; Lee 2018; Somashekhar 2018; Yu 2021; Zhou 2019). For this purpose, we determined the concordance rates of diagnostic and therapeutic recommendations for newly diagnosed cases proposed by our DSS and a certified MDT for patients with cutaneous malignant melanoma.

### Smartphone application

The smartphone application EasyOncology (EO) was developed by clinically experienced oncologists and is intended to provide evidence-based diagnostic and therapeutic recommendations for common solid cancer entities. EO’s oncological treatment algorithm “*therapy finder”* is based on a decision tree, which was developed through a systematic process with clinical experts in oncology across diverse institutions. More precisely, EO’s platform is based on current oncological guidelines, (e.g., S3-guidelines (Dkg 2020) and NCCN guidelines (Swetter 2021)), drug approval status, current publications of relevant studies, and best clinical practice from leading German cancer centers.

Frequent testing and challenging of the algorithm with real-world test cases enables identification of practice changing medical standards with subsequent corresponding adjustment of the query. Finally, frequent version updates ensure to display the latest advancements in the field of dermato-oncology.

EO was ranked top three in a worldwide comparison of 157 oncological applications in 2017 and was certified as a medical device in 2020 (Calero 2017). The software is a CE marked medical device and subject to the according regulations to ensure its security and reliability. The software relies on anonymous input without exchange of identifiable patient information via hospital intranet.

**Fig. 1:**
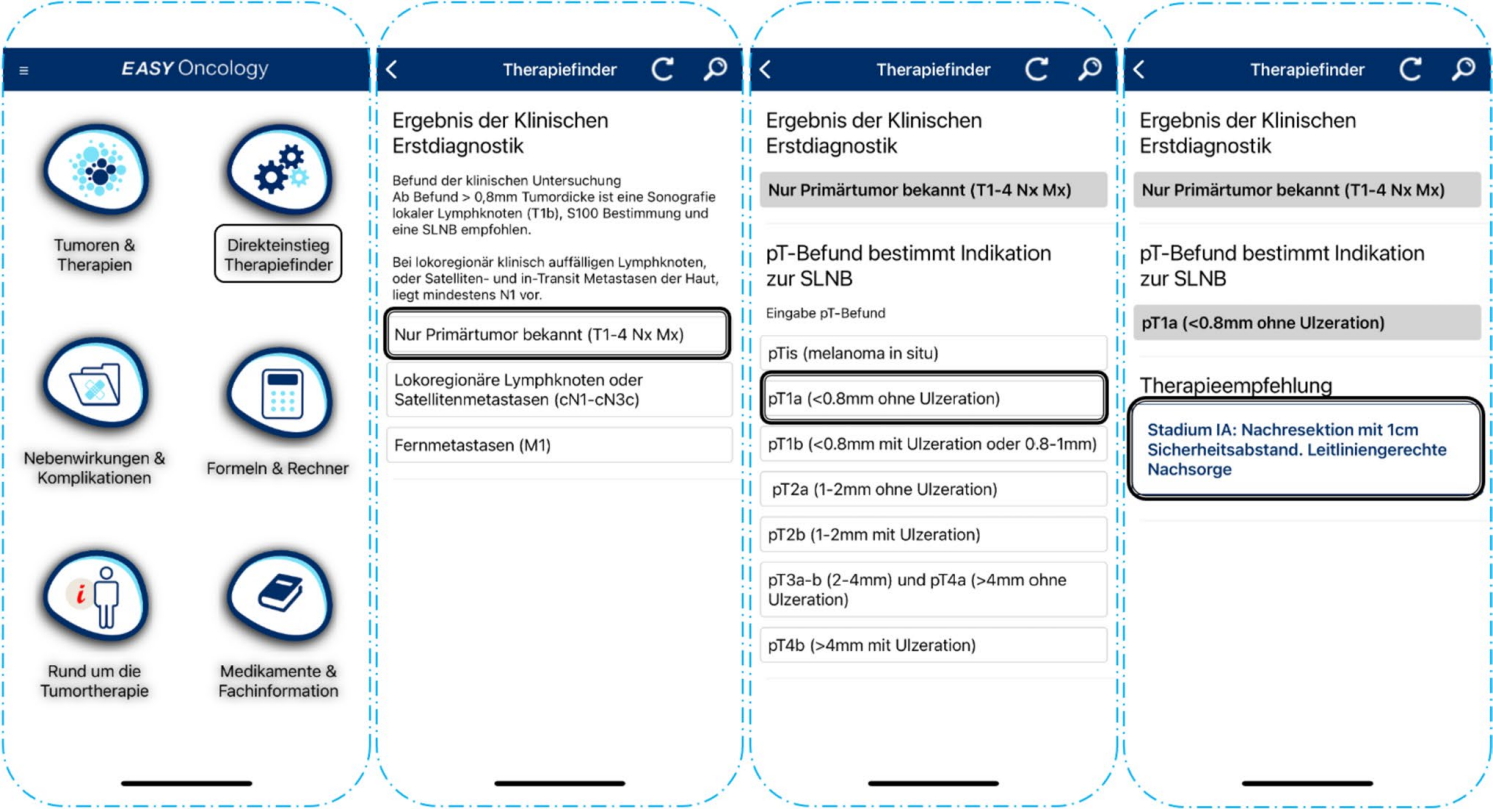
App interface of EasyOncology’s therapy finder: Stepwise diagnostic query for malignant melanoma treatment recommendation. Relevant information is requested by EO’s query algorithm to enable a treatment recommendation. In this example of primary disease, the pT-stage is at first requested. Subsequently, the algorithm query requires the Breslow thickness, which, in this example, leads to the treatment recommendation for stage IA melanoma

In the present work, EO’s *therapy finder (version 5.06)* was used to generate first-line diagnostic and therapeutic recommendations for patients with all stages of newly diagnosed cutaneous malignant melanoma. This software version displayed the 8th edition of the AJCC classification for melanoma. A graphic illustration of the App interface of EasyOncology’s *therapy finder* is depicted in Fig. 1.

The DSS query algorithm of EO’s *therapy finder* requests clinicopathologic data to generate treatment recommendations in a stepwise fashion.

The variables requested by the DSS included Breslow thickness; the presence of an ulceration (pT-stage); the histopathologic results of the sentinel lymph node biopsy (SLNB); radiologic staging information; the histopathologic results of the complete lymph node dissection (CLND); the postresection residual cancer status (R0, R1, and R2); the clinical and pathological evaluation of in-transit and satellite metastases; and the presence of important driver gene mutations (i.e., BRAF, NRAS, and cKIT).

**Fig. 2:**
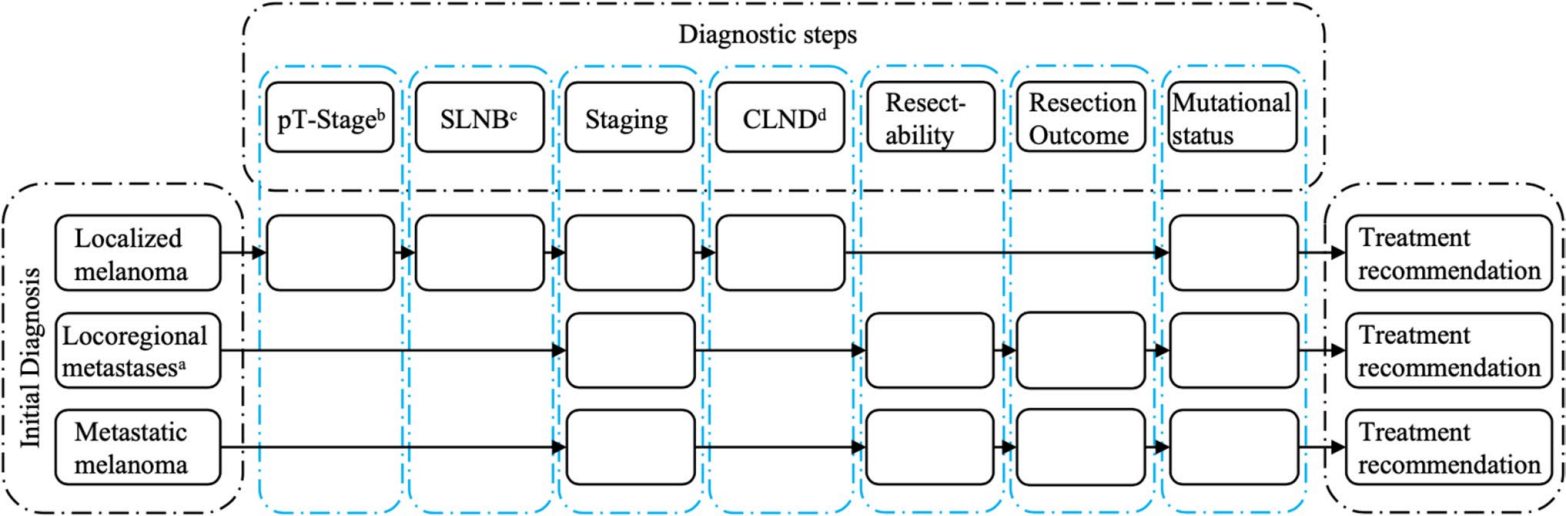
Query algorithm of EasyOncology’s therapy finder-based DSS. Depending on the selected initial diagnosis relevant diagnostic steps are requested by EO’s query algorithm until a treatment recommendation is given. Abbreviations: a: including satellite and in-transit metastases; b: determined by Breslow thickness (in mm) and presence of an ulceration; c: sentinel lymph node biopsy; d: complete lymph node dissection

The number of input variables necessary to generate a treatment recommendation depended on the complexity of each case. For simple cases (i.e., early-stage localized malignant melanoma) merely two variables are needed for DSS output, whereas more complex cases required up to five clinicopathologic variables. A simplified graphic illustration of how EO’s *therapy finder*-based DSS generates treatment recommendations is depicted in Fig. 2.

### Definition of standard cases and study inclusion criteria

According to certification criteria, all treatment cases from stage IIC upward must be presented in MDT and given recommendations are to be documented. Of note, those cases whose treatment concepts can be decided without extensive discussion can already be defined as "standard cases" when registering for the conference and thus flagged in the conference protocol. In this context, clear guideline cases, e.g., early stages that do not require extensive interdisciplinary discussion, are considered as standard cases. Since a discussion of these standard cases is not mandatory, they do not consume any time of the actual conference.

In clinical routine, these possible standard cases are often not recognized at the time of registration, for example because the registering clinician does not have sufficient clinical experience. Thus, an increase in the proportion of standard cases that were pre-answered by a DSS could relieve the tumor conference accordingly.

**Fig. 3:**
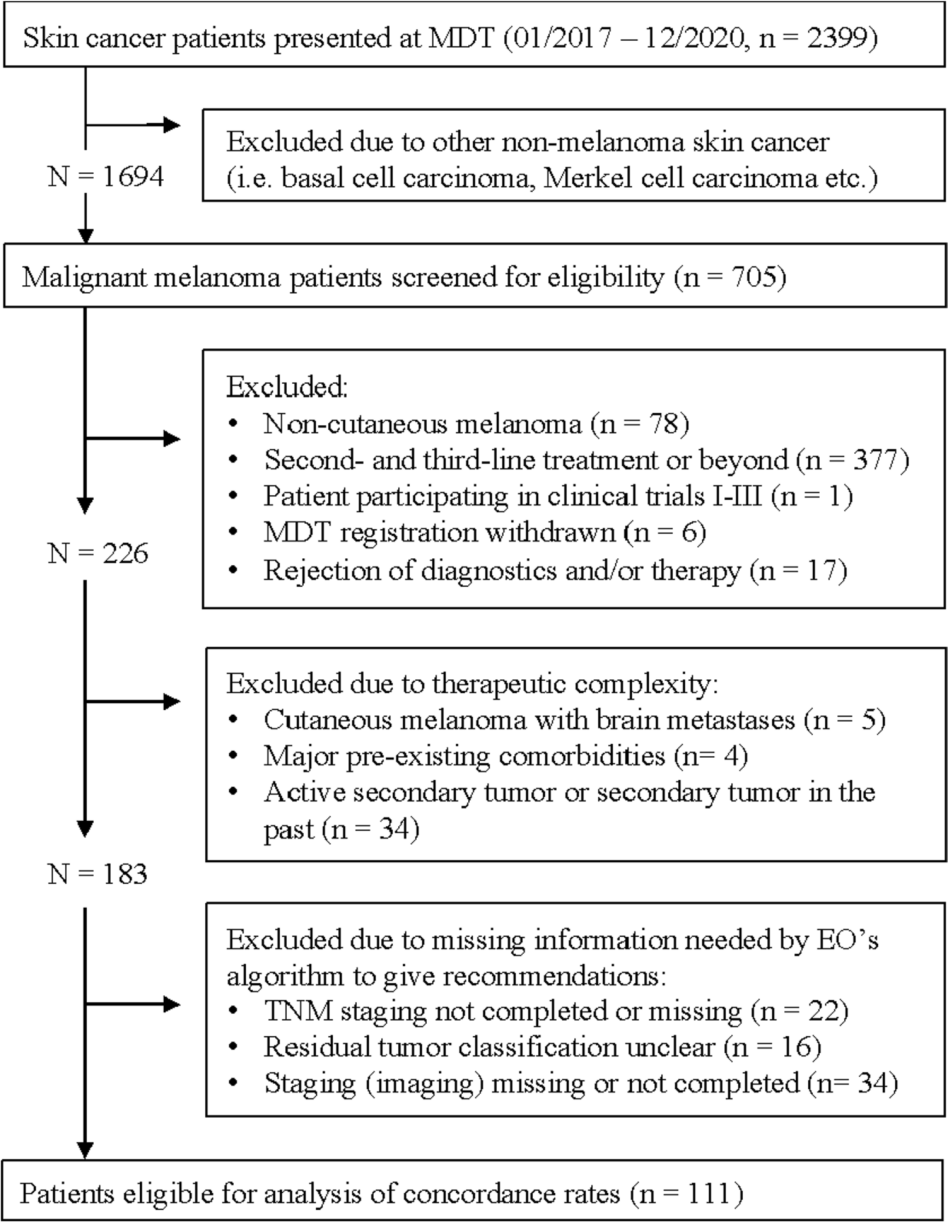
Flowchart of patient case exclusion process

In accordance with the specifications for automatic definition as a standard case, only cases without complicating factors were included in the analysis (Fig 3.). This procedure also corresponds to our approach that complicated cases should of course be discussed in the tumor board and by no means just decided digitally.

Cases with non-cutaneous melanoma (i.e., mucosal melanoma, uveal melanoma, or melanoma of unknown origin), as well as patients with relapsed disease, with secondary cancers, severe comorbidities, patients treated in clinical trials, or patients who explicitly declined diagnostic procedures (i.e., SLNB) were excluded from evaluation.

By intention, cases with brain metastases were also excluded from analysis, as stereo tactical or neurosurgical procedures should not be declared as standard cases. Lastly, we excluded those cases that lacked relevant clinicopathologic data needed as input variables by EO’s *therapy finder*.

### Patient selection and study design

Ethical approval to conduct this work was granted by the Ethics Committee of the Medical Faculty of the University of Cologne (#20-1116).

The retrospective MDT dataset evaluation initially included 2399 cases with malignant diseases of the skin who received treatment at the Department of Dermatology and Venereology, University of Cologne, between January 2017 and December 2020.

As depicted in the study inclusion flowchart (Fig 3), we first excluded all non-melanoma cases. In total, 705 melanoma cases remained that were screened for eligibility, of which 594 patient cases presented to the MDT were not suitable for analysis with EO.

Finally, MDT treatment recommendations of 111 cases that fulfilled our pre-selection criteria for standard cases were included for comparison. Hereafter, the clinical information that remained was used to generate a DSS treatment recommendation.

Treatment recommendations for each case given by MDT or DSS were compiled as response pairs. Subsequently, after blinding of decision origin, each response pair was assessed for obvious discrepancies in recommendations and accordingly classified as “concordant” or “incorrect recommendations.”

In a second independent review, an experienced dermato-oncologist analyzed each non-concordant decision pair for their quality of decisions and sub-grouped them in three categories, similar to previous publications evaluating the DSS Watson for Oncology by IBM (Choi 2019; Kim 2019; Lee 2018; Somashekhar 2018; Yu 2021; Zhou 2019):

1. If both decisions of DSS and MDT were identical, recommendations were classified as “concordant.”
2. If both decisions of “non-concordant” cases were different, but correct clinical alternatives, classification changed to "correct alternative recommendation.”
3. Case pairs were classified as "incorrect recommendation" if one of the digital or real-world recommendations was either incongruent with current best clinical practice guidelines or did not provide any recommendation, at all.

In fact*,* if using comparative cases from the past, the DSS will provide an updated treatment recommendation, thus leading to a concordance rate that only reflects the change of treatment concepts between two time points. Therefore, we assumed historic MDT treatment decisions to be correct and decided to classify this time-dependent deviation in the same group as those more actual cases with correct alternative recommendation according to best clinical standard.

As an example, for the group of "correct alternative recommendation” cases, our DSS recommended adjuvant therapy based on the mutational status, whereas, prior to 2018, MDT issued a recommendation for adjuvant interferon.

**Fig. 4:**
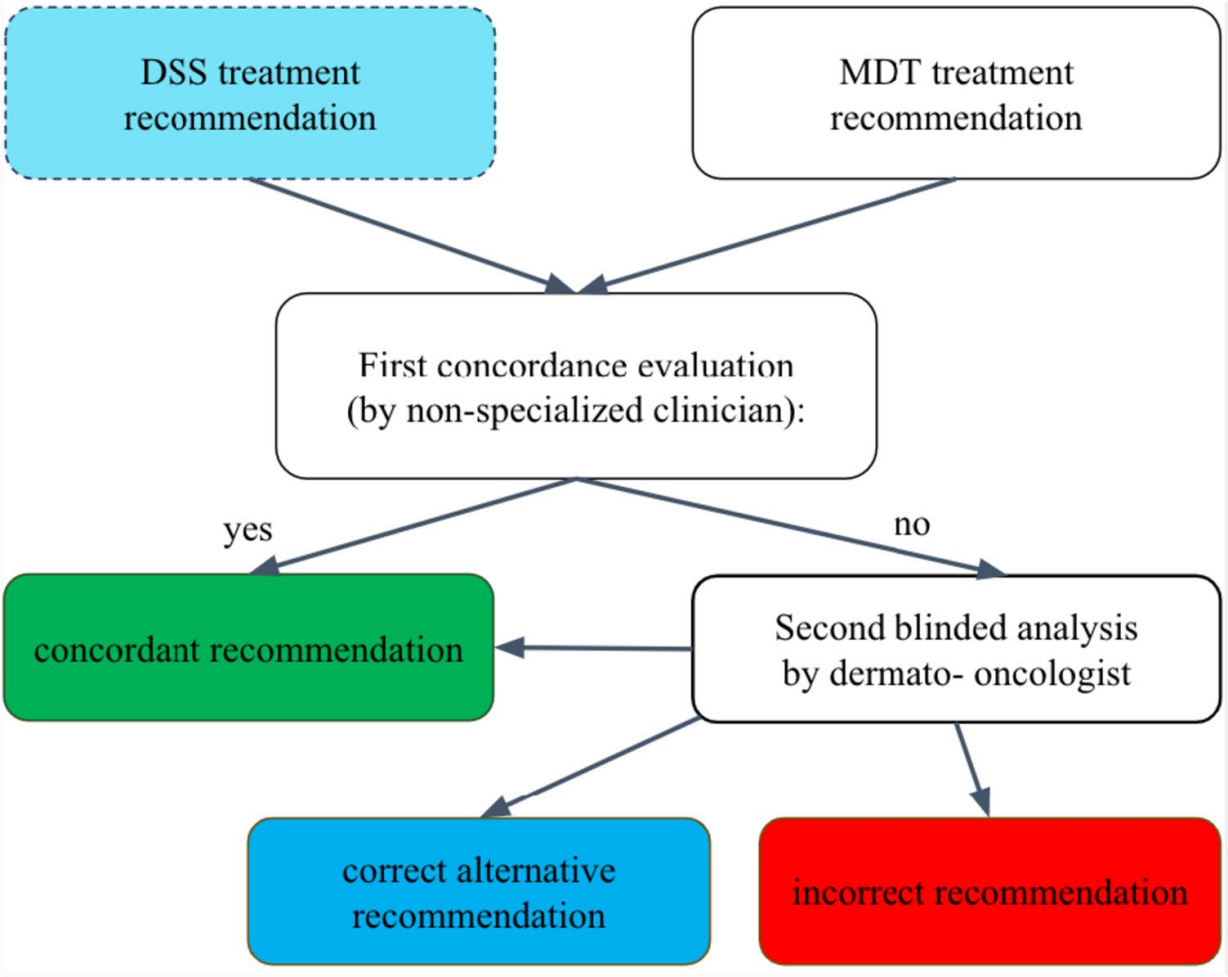
Flowchart for evaluating decision concordance of recommendations given by MDT or DSS. A first evaluation compared DSS and MDT treatment recommendations for concordance. Discordant recommendations were blinded to their origin and analyzed in detail by an dermato-oncologist, who categorized each recommendation either as “concordant recommendation,” "correct alternative recommendation" or as "incorrect recommendation"

Finally, “incorrect recommendations” cases were analyzed in detail to identify potential algorithm query errors. The evaluation process is depicted in Fig. 4.

### Data analysis and statistics

Descriptive statistics and data analysis were carried out using IBM’s statistics software SPSS version 28 and Microsoft Excel. Descriptive statistics were depicted as numbers, percentages, or median. In line with previous publications, the concordance rate was presented as a percentage agreement between DSS and MDT, i.e., $$overall concordance =\frac{concordant recommendations + correct alternative recommendations}{all recommendations}$$ (Choi 2019; Kim 2019; Lee 2018; Somashekhar 2018; Yu 2021; Zhou 2019). After assigning patients to the concordant or the non-concordant group, a Chi-squared test was used to compare categorical variables and the Mann–Whitney U test was applied to compare ordinal and numerical variables between the groups. Statistical significance was assumed if the p-value was < 0.05 for all statistical analysis. Graphics, charts, and tables were generated using SPSS, Microsoft Excel, and PowerPoint.

## Results

### Descriptive statistics

Clinicopathologic characteristics of 111 malignant melanoma patients fulfilling our predefined standard case inclusion criteria for the determination of concordance are depicted in Table 1.

**Table 1:** Clinicopathologic characteristics of malignant melanoma patients selected for concordance analysis

Clinicopathologic characteristics				n (%)	concordant	non-concordant	p-value
Malignant melanoma patients				111	108	3	
Median age (range)				62 (52–72)	59 (51–70)	57 (57)	.476
≤ 45				16 (14.4%)	16 (14.8%)	0 (0%)	.553
45–65				43 (38.7%)	41 (38.0%)	2 (66.7%)	
≥ 65				52 (46.8%)	51 (42.7%)	1 (33.3%)	
ECOG performance status, n (%)							.011*
ECOG 0				93 (83.8%)	91 (84.3%)	2 (66.7%)	
ECOG 1				9 (8.1%)	9 (8.3%)	0 (0%)	
ECOG 2				6 (5.4%)	6 (5.6%)	0 (0%)	
ECOG 3				3 (2.7%)	2 (1.9%)	1 (33.3%)	
Status at initial diagnosis, n (%)							<.001**
Localized melanoma				99 (89.2%)	98 (90.7%)	1 (33.3%)	
Regional lymph node metastases				7 (6.3%)	5 (4.6%)	2 (66.7%)	
Metastatic melanoma				5 (4.5%)	5 (4.6%)	0 (0%)	
Melanoma stage, n (%)							.871
I				3 (2.7%)	3 (2.8%)	0 (0%)	
II				19 (17.1%)	18 (16.7%)	1 (33.3%)	
III				85 (76.6%)	83 (76.9%)	2 (66.7%)	
IV				4 (3.6%)	4 (3.7%)	0 (0%)	
Histogenetic type, n (%)							.906
Superficial spreading melanoma				26 (23.4%)	26 (24.1%)	0 (0%)	
Nodular melanoma				47 (42.3%)	45 (41.7%)	2 (66.7%)	
Lentigo maligna melanoma				1 (0.9%)	1 (0.9%)	0 (0%)	
Acral lentiginous melanoma				5 (4.5%)	5 (4.6%)	0 (0%)	
Amelanotic melanoma				5 (4.5%)	5 (4.6%)	0 (0%)	
NA, other type				27 (24.3%)	26 (24.1%)	1 (33.3%)	
Sentinel lymph node biopsy, n (%)							.001**
Positive				76 (68.5%)	76 (70.4%)	0 (0%)	
Negative				14 (12.6%)	14 (13.0%)	0 (0%)	
NA				21 (18.9%)	18 (16.7%)	3 (100%)	
Gene mutation status, n (%)							.943
BRAF				9 (8.1%)	9 (8.3%)	0 (0%)	
NRAS				6 (5.4%)	6 (5.6%)	0 (0%)	
cKIT				1 (0.9%)	1 (0.9%)	0 (0%)	
wild type				6 (5.4%)	6 (5.6%)	0 (0%)	
NA				89 (80.2%)	86 (79.6%)	3(100%)	
Resection							.002**
Performed				10 (9.0%)	8 (7.4%)	2 (66.7%)	
Not performed				2 (1.8%)	2 (1.9%)	0 (0%)	
NA				99 (89.2%)	98 (90.7%)	1 (33.3%)	
Postresection residual cancer status, n (%)							<.001**
R0				8 (7.2%)	7 (6.5%)	1 (33.3%)	
R1				2 (1.8%)	1 (0.9%)	1 (33.3%)	
NA				101 (91.0%)	100 (92.6%)	1 (33.3%)	
Complete lymph node dissection, n (%)							>.999
Performed				1 (0.9%)	1 (0.9%)	0 (0%)	
Not performed				110 (99.1%)	107 (99.1%)	3 (100%)	

Values are presented as median or number (%). Concordant cases include “concordant” cases and cases defined as "correct alternative recommendation” cases

*ECOG* Eastern Cooperative Oncology Group; *NA* not available (not relevant or” pending” for EO‘s query); *R0* no residual cancer; *R1* macroscopic residual cancer removed, while margins remain positive for microscopic residual cancer. Bold type numbers indicate statistical significance

**denotes that the p-value is significant at 1% level, and * that the p-value is significant at 5% level

Median age of these patients was 62 years [interquartile range 52–72], and most patients (*n* = 93, 83.8%) had a good performance status (Eastern Cooperative Oncology Group (ECOG): 0) (<.001). At initial diagnosis, the majority of patients (*n* = 99, 89.2%) presented with localized melanoma, 7 (6.3%) presented with “regional lymph node metastases,” and 5 (4.5%) of them had “metastatic melanoma.” Ninety (81.1%) patients underwent a “sentinel lymph node biopsy” (SLNB) of whom 76 (68.5%) had at least one “positive” SLNB and 14 (12.6%) had a “negative” SLNB. A “complete lymph node dissection” was performed in 1 (0.9%) case. The number of patients with stage I, II, III, and IV melanoma was 3 (2.7%), 19 (17.1%), 85 (76.6%), and 4 (3.6%), respectively.

### Concordance rates

**Fig. 5:**
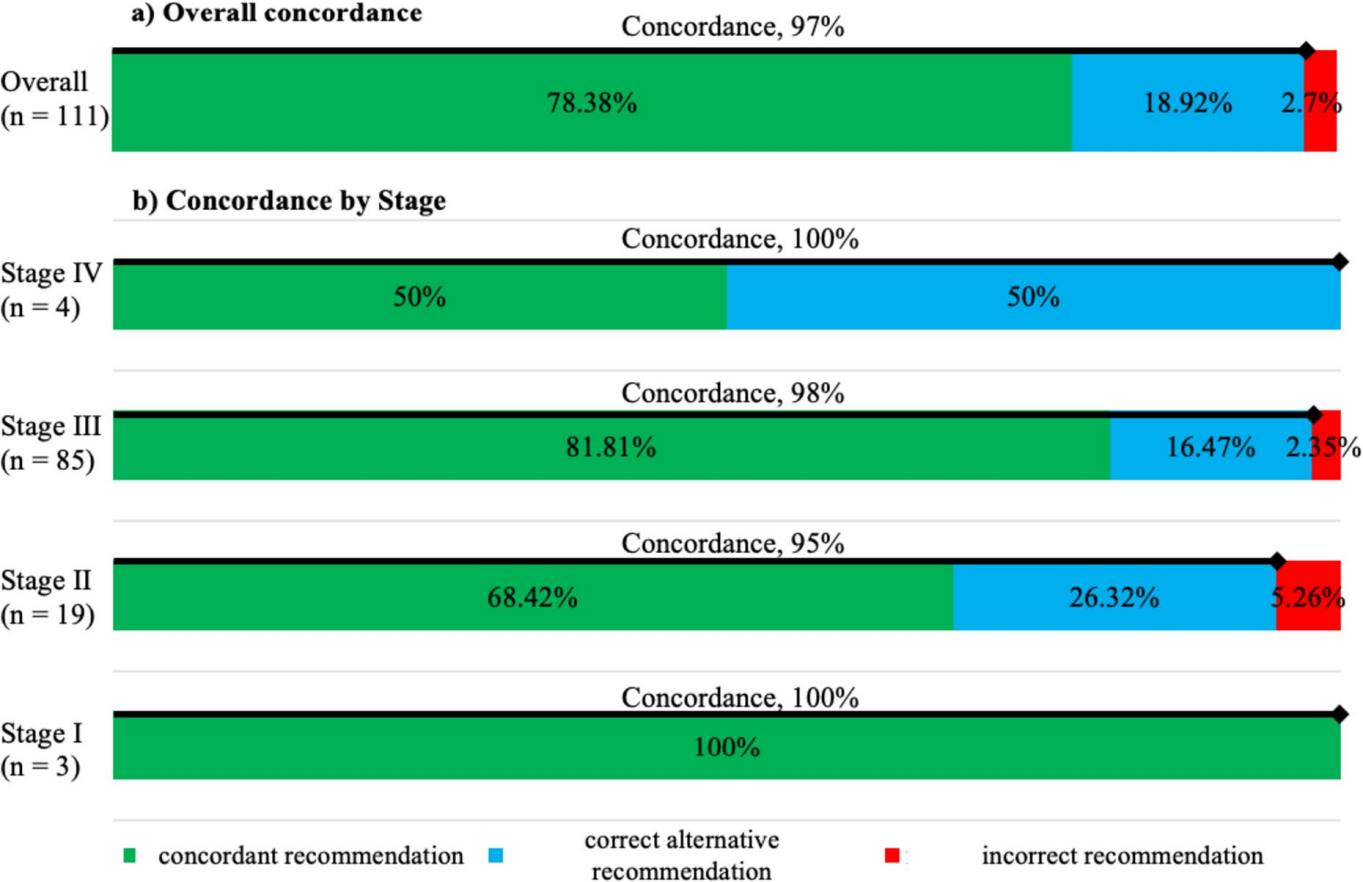
Treatment concordance between DSS and MDT. **a** Overall treatment concordance rates between the therapeutic recommendation given by the MDT and the treatment recommendation given by DSS for malignant melanoma. Overall concordance was 97%. **b** Treatment concordance rates according to malignant melanoma stages I, II, III, and IV were 100%, 95%, 98%, and 100%, respectively

In decisions regarding the optimal first-line treatment for patients with malignant melanoma, the overall concordance rate between recommendations proposed by our DSS and those given by MDT was 97%. This includes 87 (78%) “concordant” cases and 21 (19%) “correct alternative recommendation” cases (Fig. 5a). Treatment concordance rates according to malignant melanoma stages, i.e., I, II, III, and IV, were 100%, 95%, 98%, and 100%, respectively (Fig. 5b). Quality of concordance was independent of age, melanoma stage, histologic subtype, gene mutation status, and complete lymph node dissection.

### Non-concordant cases

As requested by protocol, the 3 “incorrect” cases were analyzed to identify potential systematic errors caused by our DSS decision algorithm. This independent review process was performed by an experienced dermato-oncologist. Two of these non-concordant cases with high-risk melanoma (pT4b) showed either suspicious cervical lymph nodes or a solitary pulmonary nodule in CT imaging after primary resection. Because these findings remained unclear from a diagnostic perspective, the DSS advised that both cases need to be discussed in the MDT to find an individualized solution and no therapeutic recommendation was provided. The third “incorrect” case was a 80-year-old patient who presented with cutaneous satellite metastases. In accordance with the S3 guidelines for malignant melanoma (Dkg 2020), the DSS recommended a complete surgical resection of satellite metastases and adjuvant therapy. In contrast, MDT recommended an oncolytic viral immunotherapy with talimogene laherparepvec (T-VEC). According to the reviewing dermato-oncologist, this therapy was correctly recommended by MDT due to the advanced age of the patient and the difficult resection site of the melanoma. Even though our DSS provided the correct stage-specific therapy recommendation, its recommendation was not checked for its applicability due to patient-specific factors.

## Discussion

Treatment in certified tumor centers undeniably improves the quality of oncological care, and MDTs are one of the most important quality key features. In fact, however, it is relatively unclear to what extent the tumor boards have contributed to the improvement in survival rates in the certified centers (Devitt 2013; Keating 2013; Krasna 2013; Soukup 2019; Specchia 2020).

The certification requirement to present the majority of tumor cases in MDTs has led to a noticeable increase in the number of cases to be discussed in the conferences, which could have an unfavorable effect on the quality of recommendations (Soukup 2016; Walraven 2019).

Data evaluating the extent to which MDT improve outcomes remain mixed and some studies have found a survival benefit, while others have found no such advantage. A 2019 analysis indicated that the 5 year survival rate was 15.6% higher among cases in well-organized MDT but almost 20% lower in disorganized MDT compared with no MDT (Keating 2013; Kesson 2012; Lu 2019; Stone 2020; Wong 2022).

It seems almost surprising that AI-based systems still have not become established to support decision making of MDT in routine clinical oncology. However, previous attempts to provide standardized treatment recommendations for the first-line treatment of tumor diseases using AI-based applications have shown too much uncertainty compared to the expertise of experienced oncologists. In several studies, for example, the use of Watson for Oncology could only achieve agreement rates of 12% to 93% in direct comparison with real MDT decisions (Zhou 2019; Choi 2019; Kim 2019; Lee 2018; Somashekhar 2018; Seidman 2015).

These previous AI approaches have been tested in many different tumor entities, but experience in decision support for melanoma therapy has not yet been published.

One important reason for the poor performance of AI systems is the lack of high-quality training datasets. They are available en masse as standardized data for AI systems used in image recognition, but not as regular cases in oncology care (McKinney et al. 2020; Ardila et al. 2019; Rubin 2019; Esteva et al. 2017).

In addition to the lack of well-organized and verified training data, another problem is the limited resource of experts who are initially required for the human interpretation and evaluation of the AI results.

An optimized workflow of the tumor conference is another crucial criterion for effectiveness. As requirements for an optimally structured conference, all information that is necessary for a therapy decision should first be available. In addition, those cases that require greater concentration due to their complexity should ideally be in the focus of the conference. Those medically simple cases for which clear recommendations can easily be derived from the guidelines should ideally be noted in the protocol as standard cases and only discussed optionally.

Here we see digital decision support systems as a suitable tool for structural improvement. By issuing guideline-compliant therapy recommendations at the time of conference registration, standard cases could be defined, and the conference could be unburdened accordingly.

In this respect, we see malignant melanoma as a suitable model disease for the development of decision support for tumor boards, as the melanoma treatment algorithm is considered comparatively complex.

Our dataset included 2399 cases with malignant diseases of the skin, of which 705 melanoma cases were discussed in the tumor board of the Skin Cancer Center of the University Hospital Cologne in the period from 2017 to 2020. This fits in well with the DKG annual reports of the certified skin cancer centers, which for example shows a melanoma proportion of 22.2% of all presented skin cancers for the year 2022 (Dkg 2022).

The fact that only 111 of the 705 cases with malignant melanoma could be included in the evaluation of our work is mainly due to the high proportion of recurrent diseases (377), non-cutaneous melanomas (78), and very complex disease patterns (43) that obviously cannot be declared as standard cases (see flowchart of patient case exclusion process, Fig 3). In addition, no therapy recommendation could be automatically derived in 72 cases due to insufficient information relevant for decision making.

As a result, we found three cases with divergent standard recommendations. Of these, two cases were rated as non-compliant in accordance with the protocol, as no automated recommendation was made at all, but rather a presentation to the tumor board was correctly recommended.

An important finding was the identification of a case in which the recommendation deviated due to the patient's advanced age. This implies to adapt the query algorithm to assess age in these clinical constellations so that systemic treatment can be critically discussed with the patient, especially in adjuvant treatment.

The present work has several limitations that should be considered.

As the first limitation, we only selected first-line cases and thus missed a large number of discussions on relapsed cases with first metastatic disease. In addition, certification requirements only request the presentation of melanoma cases in stages IIC and higher, leading to a low number of stage I melanoma cases.

As we included cases that were presented to MDT for the first time during the clinical course, there were few cases with initial metastatic disease. Thus, concordance rates for stage I and stage IV were calculated based on a small number of patient cases (3 and 4 patient cases, respectively). For these stages, no general conclusions can be drawn and further validation is necessary.

The second limitation derives from our strict inclusion criteria, which intended a preselection of cases that were most likely standard cases. As a consequence only 16 % of all cases were selected for detailed comparison, most of them stage II and III. If the high proportion of up to 50% stage II and III melanoma cases is taken into account, which displays the reality of MDT in the annual DKG reports, the potential to unburden MDT by DSS becomes more evident. Our evaluation also indicates 10% of cases (*n* = 72) for which the DSS was unable to issue a recommendation due to missing information. If treatment-relevant information would be obtained more systematically by a DSS at the time of conference registration, a further reduction in the burden could thus be easily derived.

As a third limitation, S3 guidelines for malignant melanoma were updated four times (DKG 2020) and numerous new therapeutics received approval during study period between 2017 and 2020. These changes in the guidelines led to deviations in the recommendations, for example on the role of interferon therapy. Of note, the presented data are based on a software (EO version 5.06) that displayed the 8th edition of the AJCC classification for melanoma and remained unchanged during the study period. Changes from 7^th^ to 8^th^ version in 2018 did not affect cases included in this study.

The development of the DSS in collaboration with the skin cancer center of the University Hospital Cologne could indicate a performance bias and the risk of overfitting. In further studies validation has to be performed by a multicentric approach. However, it is important to emphasize that the decision logic is based on the S3 guideline for malignant melanoma (Dkg 2020) and the approval status of new therapeutics that are used in best clinical practice. The continuous comparison of real world to digital decisions ensures that new treatment concepts are quickly detected, and thus, enables EO’s expert curators to integrate the latest medical standard and to appropriately adjust the query algorithm.

As a statistical limitation, the research methodology used to determine concordance rates is only of descriptive nature and describes the degree of agreement between DSS and MDT. At this time, no conclusion of the clinical benefit using DSS, such as overall survival or progression free survival of the patients, can be drawn from this approach.

The advantages we show with this work could not only provide substantial support in everyday clinical practice, but also provide a basis for the later integration of AI-based systems.

As a first advantage, the proposed principle allows an automatic structuring of the tumor conferences according to complexity and enables a quality assurance of the recommendations given by automatic comparison with the guidelines of the medical societies.

Second, the system ensures that all information necessary for a therapeutic decision is already requested at the time of registration for the conference. Otherwise, missing information often leads to postponement or only very vague recommendations such as "indication for systemic therapy."

The repetitive questioning of decision-relevant information that occurs with repeated use leads to the third advantage, the teaching effect. This could also be reinforced by the sense of satisfaction that comes from having received a (digital) treatment recommendation.

Not least, another advantage is that the type of recommendation matching described above generates training data for the future integration of AI systems. As said before, a machine learning tool can only ever be as good as the data available for training and the trainers who evaluate the AI results.

## Summary

It must be underlined again that the aim of our work is not to provide digital recommendations for all questions addressed to multidisciplinary tumor boards or to replace clinical experience. However, we see the need to relieve the time burden of these critical conferences so that the participants can focus their expertise on the more complex tumor cases. Our results suggest that this automated approach would allow a more concentrated and detailed discussion of complex tumor cases, on which the valuable expertise of the board members should be focused. In addition, the implementation of the presented algorithm in the routine software of tumor boards could provide the basis for transparent and comparable quality management.

### Perspective

The principle of digital decision support described in this paper, whose algorithmic query is based on a decision tree, may seem unspectacular at first glance against the background of current AI development. However, it is currently the most suitable way to make recommendations for clinical situations that are already defined by clear therapeutic standards and guidelines. These commonly accepted therapeutic recommendations are based, among other things, on the approval status and availability of the therapeutic agents, as well as some clinical and structural aspects which are not evidence-based per se. It should therefore come as no surprise that the recommendations made by AI-based systems for well-defined standard clinical situations often do not correspond to standard practice. At this point, it should be considered that the AI recommendations could well be a better therapeutic choice, though this cannot be proven due to the AI black box effect described above.

Accordingly, it is to be expected that AI applications will initially realize their full potential in complex clinical constellations of advanced cancer diseases in which clinical standards do not exist.

However, this requires the availability of sufficient training data generated under the requirements of a defined healthcare system in routine care. This is precisely the data that are currently lacking, however, training data from other countries and healthcare systems cannot be used without hesitation.

The intended integration of query algorithms into the organizational software of tumor boards offers an important cornerstone here. The data collected on standard situations in oncological care can serve as the necessary training data for the planned integration of suitable AI models.

However, there is still a long way to go before AI delivers such impressive results in decision making as we are currently seeing with image-supported AI systems.

## Data Availability

All data contained in the manuscript as well as the primary metadata are based on the tumor board documentation of the University Hospital Cologne. The data were evaluated in a pseudonymized form in compliance with data protection guidelines and can be assigned to the real cases by the treating physicians who are permitted to inquire about them.

## References

[CR1] Amaria RN, Postow M, Burton EM et al (2022) Neoadjuvant relatlimab and nivolumab in resectable melanoma. Nature 611(7934):155–60. 10.1038/s41586-022-05368-8 36289334 10.1038/s41586-022-05368-8 PMC9607737

[CR2] Ardila D, Kiraly AP, Bharadwaj S et al (2019) End-to-end lung cancer screening with three-dimensional deep learning on low-dose chest computed tomography. Nat Med 25(6):954–61. 10.1038/s41591-019-0447-x 31110349 10.1038/s41591-019-0447-x

[CR3] Beckmann MW, Brucker C, Hanf V et al (2011) Quality assured health care in certified breast centers and improvement of the prognosis of breast cancer patients. Onkologie 34(7):362–7. 10.1159/000329601 21734422 10.1159/000329601

[CR4] Bierbaum M, Rapport F, Arnolda G et al (2020) Clinicians’ attitudes and perceived barriers and facilitators to cancer treatment clinical practice guideline adherence: a systematic review of qualitative and quantitative literature. Implement Sci 15(1):39. 10.1186/s13012-020-00991-3 32460797 10.1186/s13012-020-00991-3 PMC7251711

[CR5] Bierbaum M, Rapport F, Arnolda G et al (2023) Rates of adherence to cancer treatment guidelines in Australia and the factors associated with adherence A systematic review. Asia Pac J Clin Oncol. 10.1111/ajco.13948.10.1111/ajco.13948 36881529 10.1111/ajco.13948

[CR6] Bungartz KD, Lalowski K, Elkin SK (2018) Making the right calls in precision oncology. Nat Biotechnol 36(8):692–6. 10.1038/nbt.4214 30080824 10.1038/nbt.4214

[CR7] Calero JJ, Oton LF, Oton CA (2017) Apps for Radiation Oncology A Comprehensive Review. Transl Oncol 10(1):108–14. 10.1016/j.tranon.2016.08.008 28164839 10.1016/j.tranon.2016.08.008 PMC5300110

[CR8] Chen Y, Elenee Argentinis JD, Weber G (2016) IBM Watson: How Cognitive Computing Can Be Applied to Big Data Challenges in Life Sciences Research. Clin Ther 38(4):688–701. 10.1016/j.clinthera.2015.12.001 27130797 10.1016/j.clinthera.2015.12.001

[CR9] Choi YI, Chung JW, Kim KO et al (2019) Concordance Rate between Clinicians and Watson for Oncology among Patients with Advanced Gastric Cancer: Early, Real-World Experience in Korea. Can J Gastroenterol Hepatol 2019:8072928. 10.1155/2019/8072928 30854352 10.1155/2019/8072928 PMC6377977

[CR10] Devitt B, Philip J, McLachlan SA (2013) Re: Tumor boards and the quality of cancer care. J Natl Cancer Inst 105(23):1838. 10.1093/jnci/djt311 24187010 10.1093/jnci/djt311

[CR11] DKG (2020) Leitlinienprogramm Onkologie (Deutsche Krebsgesellschaft, Deutsche Krebshilfe, AWMF): Diagnostik, Therapie und Nachsorge des Melanoms, Langversion 3.3, 2020, AWMF Registernummer: 032/024OL http://www.leitlinienprogramm-onkologie.de/leitlinien/melanom/. „Accessed“14.09.2022

[CR12] DKG (2019) Nationales Zertifizierungsprogramm Krebs. Erhebungsbogen für Onkologische Spitzenzentren und Onkologische Zentren Deutsche Krebsgesellschaft und Deutsche Krebshilfe https://www.krebsgesellschaft.de/zertdokumente.html. „Accessed“ 14.09.2022

[CR13] DKG (2022) DKG Jahresbericht der zertifizierten Hautkrebszentren. Kennzahlenauswertung 2022 /Auditjahr 2021 / Kennzahlenjahr 2020.

[CR14] Esteva A, Kuprel B, Novoa RA et al (2017) Dermatologist-level classification of skin cancer with deep neural networks. Nature 542(7639):115–8. 10.1038/nature21056 28117445 10.1038/nature21056 PMC8382232

[CR16] Heins MJ, de Jong JD, Spronk I et al (2017) Adherence to cancer treatment guidelines: influence of general and cancer-specific guideline characteristics. Eur J Public Health 27(4):616–20. 10.1093/eurpub/ckw234 28013246 10.1093/eurpub/ckw234

[CR17] IQVIA (2022). Institute I https://www.iqvia.com/insights/the-iqvia-institute/reports/global-oncology-trends-2022 „Accessed“ 10.06.2022

[CR18] Jalil R, Soukup T, Akhter W et al (2018) Quality of leadership in multidisciplinary cancer tumor boards: development and evaluation of a leadership assessment instrument (ATLAS). World J Urol 36(7):1031–8. 10.1007/s00345-018-2255-1 29502300 10.1007/s00345-018-2255-1

[CR19] Keating NL, Landrum MB, Lamont EB et al (2013) Tumor boards and the quality of cancer care. J Natl Cancer Inst 105(2):113–21. 10.1093/jnci/djs502 23274388 10.1093/jnci/djs502 PMC4481638

[CR20] Kesson EM, Allardice GM, George WD et al (2012) Effects of multidisciplinary team working on breast cancer survival: retrospective, comparative, interventional cohort study of 13 722 women. Bmj 344:e2718. 10.1136/bmj.e2718 22539013 10.1136/bmj.e2718 PMC3339875

[CR21] Kim EJ, Woo HS, Cho JH et al (2019) Early experience with Watson for oncology in Korean patients with colorectal cancer. PLoS One 14(3):e0213640. 10.1371/journal.pone.0213640 30908530 10.1371/journal.pone.0213640 PMC6433269

[CR22] Kim M, Kim BH, Kim JM et al (2019) Concordance in postsurgical radioactive iodine therapy recommendations between Watson for Oncology and clinical practice in patients with differentiated thyroid carcinoma. Cancer 125(16):2803–9. 10.1002/cncr.32166 31216369 10.1002/cncr.32166

[CR23] Kowalski C, Graeven U, von Kalle C et al (2017) Shifting cancer care towards Multidisciplinarity: the cancer center certification program of the German cancer society. BMC Cancer 17(1):850. 10.1186/s12885-017-3824-1 29241445 10.1186/s12885-017-3824-1 PMC5731059

[CR24] Kowalski C, Sibert NT, Breidenbach C et al (2022) Outcome Quality After Colorectal Cancer Resection in Certified Colorectal Cancer Centers—Patient-Reported and Short-Term Clinical Outcomes. Dtsch Arztebl Int. 10.3238/arztebl.m2022.0325 36321583 10.3238/arztebl.m2022.0325 PMC10074266

[CR25] Krasna M, Freeman RK, Petrelli NJ (2013) Re: Tumor boards and the quality of cancer care. J Natl Cancer Inst 105(23):1839–40. 10.1093/jnci/djt313 24187012 10.1093/jnci/djt313

[CR26] Lee WS, Ahn SM, Chung JW et al (2018) Assessing Concordance With Watson for Oncology, a Cognitive Computing Decision Support System for Colon Cancer Treatment in Korea. JCO Clin Cancer Inform 2:1–8. 10.1200/cci.17.00109 30652564 10.1200/CCI.17.00109

[CR27] Letzen B, Wang CJ, Chapiro J (2019) The Role of Artificial Intelligence in Interventional Oncology: A Primer. J Vasc Interv Radiol 30(1):38-41.e1. 10.1016/j.jvir.2018.08.032 30528289 10.1016/j.jvir.2018.08.032

[CR29] Lu J, Jiang Y, Qian M et al (2019) The Improved Effects of a Multidisciplinary Team on the Survival of Breast Cancer Patients: Experiences from China. Int J Environ Res Public Health. 10.3390/ijerph17010277 31906051 10.3390/ijerph17010277 PMC6982185

[CR30] Luke JJ, Rutkowski P, Queirolo P et al (2022) Pembrolizumab versus placebo as adjuvant therapy in completely resected stage IIB or IIC melanoma (KEYNOTE-716): a randomised, double-blind, phase 3 trial. Lancet 399(10336):1718–29. 10.1016/s0140-6736(22)00562-1 35367007 10.1016/S0140-6736(22)00562-1

[CR31] McKinney SM, Sieniek M, Godbole V et al (2020) International evaluation of an AI system for breast cancer screening. Nature 577(7788):89–94. 10.1038/s41586-019-1799-6 31894144 10.1038/s41586-019-1799-6

[CR32] Modabber A, Schick D, Goloborodko E et al (2021) Impact of quality certification of multidisciplinary head and neck tumor centers. Cost Eff Resour Alloc 19(1):20. 10.1186/s12962-021-00273-9 33827599 10.1186/s12962-021-00273-9 PMC8028776

[CR33] Narang J, Hue JJ, Bingmer K et al (2021) Sentinel lymph node biopsy guideline concordance in melanoma: Analysis of the National Cancer Database. J Surg Oncol 124(4):669–78. 10.1002/jso.26565 34109633 10.1002/jso.26565

[CR35] Rubin R (2019) Artificial Intelligence for Cervical Precancer Screening. Jama 321(8):734. 10.1001/jama.2019.0888 30806677 10.1001/jama.2019.0888

[CR36] Schmidt C (2017) MD Anderson Breaks With IBM Watson Raising Questions About Artificial Intelligence in Oncology. J Natl Cancer Inst. 10.1093/jnci/djx113 30053147 10.1093/jnci/djx113

[CR37] Schmitt J, Schoffer O, Klinkhammer-Schalke M, Bobeth C, Roessler M, et al (2022) Wirksamkeit der Versorgung in onkologischen Zentren(WiZen)–Erkenntnisse zur Ergebnisqualität und Erfolg des Datenlinkage AOK „Accessed“ 20.08.23

[CR38] Seidman AD, Pilewskie ML, Robson ME et al (2015) Integration of multi-modality treatment planning for early stage breast cancer (BC) into Watson for Oncology, a Decision Support System: Seeing the forest and the trees. J Clin Oncol 33(15):e12042. 10.1200/jco.2015.33.15_suppl.e12042

[CR39] Somashekhar SP, Sepúlveda MJ, Puglielli S et al (2018) Watson for Oncology and breast cancer treatment recommendations: agreement with an expert multidisciplinary tumor board. Ann Oncol 29(2):418–23. 10.1093/annonc/mdx781 29324970 10.1093/annonc/mdx781

[CR40] Soukup T, Petrides KV, Lamb BW et al (2016) The anatomy of clinical decision-making in multidisciplinary cancer meetings: A cross-sectional observational study of teams in a natural context. Medicine (Baltimore) 95(24):e3885. 10.1097/md.0000000000003885 27310981 10.1097/MD.0000000000003885 PMC4998467

[CR41] Soukup T, Lamb BW, Weigl M et al (2019) An Integrated Literature Review of Time-on-Task Effects With a Pragmatic Framework for Understanding and Improving Decision-Making in Multidisciplinary Oncology Team Meetings. Front Psychol 10:1245. 10.3389/fpsyg.2019.01245 31354555 10.3389/fpsyg.2019.01245 PMC6634259

[CR42] Specchia ML, Frisicale EM, Carini E et al (2020) The impact of tumor board on cancer care: evidence from an umbrella review. BMC Health Serv Res 20(1):73. 10.1186/s12913-020-4930-3 32005232 10.1186/s12913-020-4930-3 PMC6995197

[CR43] Stone E, Rankin N, Currow D et al (2020) Optimizing lung cancer MDT data for maximum clinical impact-a scoping literature review. Transl Lung Cancer Res 9(4):1629–38. 10.21037/tlcr.2020.01.02 32953537 10.21037/tlcr.2020.01.02 PMC7481624

[CR44] Swetter SM, Thompson JA, Albertini MR et al (2021) NCCN Guidelines® Insights Melanoma Cutaneous Version 22021. J Natl Compr Canc Netw 19(4):364–76. 10.6004/jnccn.2021.0018 33845460 10.6004/jnccn.2021.0018

[CR46] Walraven JEW, Desar IME, van der Hoeven JJM et al (2019) Analysis of 105.000 patients with cancer: have they been discussed in oncologic multidisciplinary team meetings? A nationwide population-based study in the Netherlands. Eur J Cancer 121:85–93. 10.1016/j.ejca.2019.08.007 31563730 10.1016/j.ejca.2019.08.007

[CR47] Wolff KD, Rau A, Ferencz J et al (2017) Effect of an evidence-based guideline on the treatment of maxillofacial cancer: A prospective analysis. J Craniomaxillofac Surg 45(3):427–31. 10.1016/j.jcms.2016.12.013 28108238 10.1016/j.jcms.2016.12.013

[CR48] Wong BO, Blythe JA, Wu A et al (2022) Exploration of Clinician Perspectives on Multidisciplinary Tumor Board Function Beyond Clinical Decision-making. JAMA Oncol 8(8):1210–2. 10.1001/jamaoncol.2022.1763 35653129 10.1001/jamaoncol.2022.1763 PMC9164116

[CR49] Yu SH, Kim MS, Chung HS et al (2021) Early experience with Watson for Oncology: a clinical decision-support system for prostate cancer treatment recommendations. World J Urol 39(2):407–13. 10.1007/s00345-020-03214-y 32335733 10.1007/s00345-020-03214-y

[CR50] Zhou N, Zhang CT, Lv HY et al (2019) Concordance Study Between IBM Watson for Oncology and Clinical Practice for Patients with Cancer in China. Oncologist 24(6):812–9. 10.1634/theoncologist.2018-0255 30181315 10.1634/theoncologist.2018-0255 PMC6656482

